# Beyond Query-Oriented Highlighting: Investigating the Effect of Snippet Text Highlighting in Search User Behavior

**DOI:** 10.1155/2018/7836969

**Published:** 2018-12-04

**Authors:** Hui Zhang

**Affiliations:** State Key Laboratory of Intelligent Technology and Systems, Tsinghua National Laboratory of Information Science and Technology, Department of Computer Science and Technology, Tsinghua University, Beijing 100084, China

## Abstract

Search users rely on result captions including titles, snippets, and URLs to decide whether they should read and click a particular result or not. Snippet usually serves as a query-dependent summary of its corresponding landing page and is therefore treated as one of the most important factors in search interaction process. Although there exist many efforts in improving snippet generation algorithms and incorporating more powerful interaction functions into snippets, little attention is paid to the effect of text highlighting in user behaviors. The highlighting of query terms in search snippets has been regarded as a matter of course and whether there exists a better way in snippet text highlighting remains uninvestigated. In this paper, we try to find out whether the default strategy of highlighting query terms employed by most commercial search engines is the best for search users. Through carefully designed experiments, we show that the retrieval efficiency can be affected by different term-highlighting strategies without changes in snippet contents. We also propose an automatic method which adopts CRF to learn to highlight terms based on word embedding, Wikipedia, and snippet content information. Experimental results show that the proposed method could predict highlighted terms selected by crowd workers with moderate performance.

## 1. Introduction

For most commercial search engines, although many novel forms of search results (e.g., verticals [1, 2], cards [3, 4], knowledge graphs [5], and direct answers [6]) have been incorporated into result lists, the major parts of results are still in the traditional form which contains title, snippets, and URLs. Search users rely on this caption information to decide whether they should click on the result and read the content of the landing page. Therefore, the organization of result caption information, especially the generation of snippets, is closely related with user's search interaction process and has been one of the major concerns in search engine UI studies [7–12]. Most of these existing studies investigate the appropriate presentation styles of snippets for search users such as length [7, 13] and readability [8, 14]. They also try to generate better snippets to improve search user satisfaction or search efficiency with improvement in both content summarization [6] and interactive functions [15]. Although there are a small number of works which focus on the impact of snippet highlighting in search [16], most works just assume that query term highlighting is the natural way in search UI designing. Little work has been done on investigating whether it is the best way to help search users to locate relevant information and how we can improve this simple yet important strategy in search result presentations.

**Figure 1:**
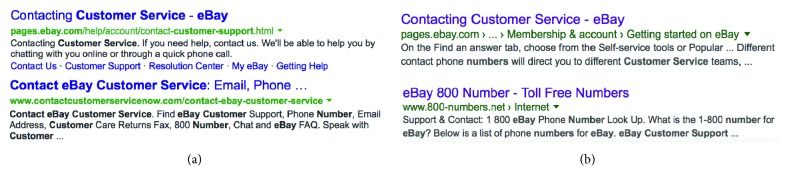
Example search results with different highlighted terms from Bing (a) and Google (b) for the query “eBay customer service number.”

In information retrieval researches, text highlighting refers to altering the appearance of portions of text in order to make them more visually salient [17]. In Web search scenarios, text highlighting is usually in the form of highlighting query terms (or their synonyms). This kind of query term highlighting strategy is adopted in both snippet contents and result titles (see Figure 1 for some examples). According to both manual experimental results [16] and eye-tracking studies [10], query term highlighting can help draw the search user's attention to the results that are most likely to be relevant to the query and even change their allocation of attention to some lower-ranked results on SERPs.

Considering the great efforts researchers have spent on generating better result snippets, we still know little about the effect of different term highlighting strategies on users' search behavior. Search engine result pages (SERPs) have long evolved from a linear list of homogeneous results to a much more heterogenous combination of information units; while the term highlighting strategies remain almost the same since the age of “ten blue links.” Currently, some search engines try to also highlight terms that are not directly from user queries but highly related with query terms (such as the term “support” in the lower example of Figure 1(b)). However, which kind of extraterms besides query terms should be highlighted and how this kind of revised highlighting strategy changes user behavior still remain uninvestigated as far as we know.

The Query Term Highlighting (QTB) strategy has been adopted since the early stage of commercial search engines. It supposes that the snippets with many query term matches may represent more relevant documents and should be paid more attention. The intuition is simple but effective at least for the early search users. However, both search tasks and web information sources have evolved and are quite different from the early stage of web and web search [18]. The increasing needs of exploratory [19], dynamic [20], or diversified [21–23] search tasks create a large gap between the current query content and user's actual information needs. Meanwhile, the redundancy of web information sources has also introduced search results which may contain many matching query terms but little key information. From Figure 1, we can see four example results from Google and Bing for the query “eBay customer service number.” The higher-ranked results in both Figures 1(a) and 1(b) are from eBay's official website and contain valuable information about how to contact the customer service team, while the lower-ranked results in Figures 1(a) and 1(b) are from two not so trustworthy sites which claim to contain eBay's customer service information. The lower-ranked Google result even claim to provide eBay's 800 toll-free number (which is not possible because eBay does not provide such services (http://pages.ebay.com/help/account/contact-customer-support.html)). However, the lower-ranked result each has much more highlighted terms than the corresponding higher-ranked one (11 vs 1 for Figure 1(a) and 9 vs 3 for Figure 1(b)). It means that the users may be misled by the highlighted query terms and choose results that they are not likely to prefer.

From the above examples, we can see that the highlighting strategy that focuses on query terms (and their synonyms) may not be so reliable in contemporary search environment. We therefore try to investigate into the effect of term highlighting strategies in search behavior and see how we can find a better way to highlight terms to help users find useful information more effectively. The key research questions we want to investigate in this paper include the following: (RQ1) Which snippet terms should be highlighted to improve users' search experience? (RQ2) Which kind of highlighting strategy should be adopted: should we highlight longer continuous phrases or shorter isolated terms? (RQ3) Can we automatically suggest highlighted terms for a given SERP based on the answers to RQ1 and RQ2?

To shed light on these research questions, at first, we organize a crowdsourcing effort to annotate the terms that help users to judge results' usefulness on SERPs (Section 3). By this means, we want to generate an ideal list of highlighted terms for each result and use it as the ground truth in latter studies. After that, we perform a series of user behavior studies to compare users' different behavior signals (including both click-through and eye movement behaviors) under different term highlighting settings (Section 4). Especially, we examine whether users' search experiences benefit from the ideal highlighted term list. Finally, we try to propose a sequence labeling method which aims to select terms to be highlighted with the help of information extracted from Wikipedia, word embedding, and synonym dictionaries (Section 5). We also test the effectiveness of the proposed method with practical users' behavior signals (Section 6).

Our contributions in this paper are three-fold: (1) To our best knowledge, this is the first attempt to investigate the effect of term highlighting strategies other than query term highlighting in user's search interaction process. (2) Through comparison in users' click-through and eye movement behaviors under different term highlighting settings, we show that search users benefit more from highlighted terms selected by crowdsourcing workers than simply matching query terms. (3) We formalize the term highlighting task as a supervised sequence labeling problem and adopt conditional random field (CRF) methods to select highlighted terms based on both content-based and position-based features.

## 2. Related Work

Three lines of research are related to the work we describe in this article: (1) effect of snippet content and presentation styles in search user behavior, (2) snippet generation algorithms and corresponding evaluation methods, and (3) the effect of highlight terms in searching interaction process.

Joachims et al. pointed out that because users are known to be biased towards clicking documents with higher rankings, if a document had relatively low ranking, the snippet of the document must include compelling information that prompts the click [24]. The quality of the snippet has a strong effect on the ability of the searcher to judge the relevance of the document. Turpin et al. investigated how accounting for summary judgment stage can alter IR systems evaluation and comparison results [25]. Even the most relevant document is unlikely to be clicked if the snippet is uninformative or misleading.

Most of the work on automatic summary generation is about how to best formulate and display a summary. Several researchers have experimented with models in the sentence selection and summary length [11, 13, 26]. White et al. experimented with different sentence selection methods, including giving more weight to sentences that contained query terms along with text formatting (e.g., highlight face or italics) [12]. This method ignores the context information of a sentence. Varadarajan and Hristidis presented a method to create a query specific snippet by identifying the most query-relevant fragments and then combining them using a graph of document structure [11]. Cutrell and Guan compared search summaries of varying length, which found that adding more information to the snippet significantly improved the performance of information tasks but degraded the performance of navigational tasks [26].

The readability of snippet is an important indicator of document relevance, which was associated with receiving significantly more clicks in a query log [25]. Few varied summaries of search results along several dimensions, finding that text choppiness and sentence truncation had negative effects and genre cues had positive effects [27]. Kanungo and Orr found that some features (a large percentage of capital letters, punctuation, stopwords, and a large number of characters per word) had negatively influenced readability. In addition, the study shows that salient items can influence the readability of snippet [8]. Given this, Query terms highlighting has become a common method in today's major search engines. Kickmeier and Albert showed that the density of salient items had a clear impact on response time and answer accuracy in search tasks [28].

Human eyes are very receptive to different brightness within a text body. Text highlighting aims to change the appearance of the text in order to make them more visually salient, or “eye-catching.” The display of snippet with Color Highlighting of query terms helps to draw the searcher's attention, which has been found to be a useful feature for user experience in information access [29–33]. Few on the other hand, told us that a lot of text highlighting can reduce the ability of visual recognition [27, 34]. Snippets of SERP need to present important information clearly, precisely, and without extraneous or distracting clutter.

Most snippets are, in fact, manually crafted summaries from third-party sites (such as ODP2 descriptions) or from META field of the original HTML page. The role of snippet is sometimes referred to as the document agent that intends to help the user to understand the primary object and measure the degree of relevance of search task to the original retrieved document [22, 35]. The query terms of highlight snippet reflect the corresponding web pages which are most likely relevant to the query and show how close the query terms appear in the document [36]. At the same time, the search summary also contains a lot of nonquery words, which may also have a close relationship with the relevance of search task to the document. Furthermore, in some cases, summaries can provide the user with required information in situ (e.g., factoid questions). At present, there is no work taking into account the comprehensive role of the query words, nonquery words, factoid questions, and highlight density. In this paper, we study the effect of term highlighting strategies of snippet in user's search interaction process.

## 3. Data Collection

In this section, we describe the data collection process in our work, including a crowdsourcing effort to collect highlighted terms, three strategies for generating an oracle highlighted term list, experiment setups for collecting users' search interaction data as well as explicit feedback, and result relevance annotations.

### 3.1. Crowdsourcing

To study the effectiveness of different snippet term highlighting strategies, we carry out a crowdsourcing effort to collect the highlighted terms list. We select 24 search queries from the NTCIR IMine task [37], as shown in Table 1. We write detailed task explanations for 24 queries to avoid ambiguity, among which there are 4 navigational search tasks (NA), 16 informational search tasks (IN), and 4 transactional search tasks (TR), some of which are is shown in Table 2. We have used the questionnaire platform (https://www.wjx.cn/) to the consistency of the query and its task description.

**Figure 2:**
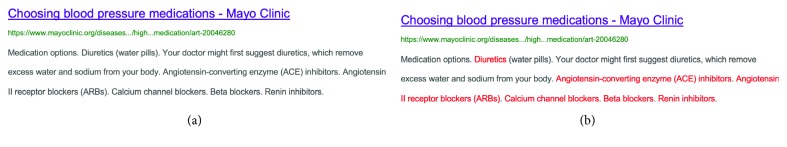
Example search results with removed highlighting terms from Google (a) and an annotated user (b) for the same query and snippet.

For each search task, we fix the query and results to ensure the consistency of our data. The search results were crawled from Google search engine and only top 10 organic results are retained. Vertical results and advertisements are excluded because they may affect user engagement [38]. We remove the original highlighted snippet terms from the original snippet to form a summary of the uniform font and color, as shown in Figure 2(a). And we invite users to highlight terms through a crowdsourcing platform (https://www.wjx.cn/) and required them to select 1∼5 snippet words or phrases (the words or phrases highlighted by users may are very long, which may consist of several terms) to be highlighted for each snippet result. The snippet of highlighting words annotated by one user is shown in Figure 2(b). We recruited 10 search users in total, who are required to have at least five years of search engine usage experience. Each user completed the 24 annotation tasks (10 results each task) and was paid 4 US dollars. In this way, we get highlighted terms from 10 users for each specific snippet result, which forms the corresponding snippet result's highlighted term list.

The users' highlighted terms reflect an interesting phenomenon, which is the users care far beyond the original query terms. For example, users highlight “early stage of AIDS” when searching “AIDS skin symptoms” and they highlight “configuration,” “price” when searching “TOYOTA REIZ.” Such terms are not query terms but can provide abundant semantic information. Instant answers are also very helpful; for example, all users during the crowdsourcing process highlight the term “October 24, 2003” when the search query is “Song Mei-ling's date of death.” Such phenomenon inspires us to develop a more reasonable highlighting strategy rather than simply make the query terms highlighted. We will make more detailed analysis in Sections 4 and 5.

### 3.2. Highlighting Strategies

To study the effect of highlighted snippet terms in user behavior, we propose three different highlighting strategies besides the original query terms highlighting method based on the highlighted terms list obtained by crowdsourcing process:

- *Original Highlighting Strategy (S1)*: This is the original query terms highlighting strategy adopted by Google and other commercial search engines. This strategy considers that the query word is a correlation indicator. The more query words, the higher the correlation.
- *Reduced Highlighting Strategy (S2)*: For each snippet result, we select to highlight the longest three query words phrases. If there exist snippet results which have fewer than 3 highlighted words, we just make all the words in its highlighted term list highlighted. With this strategy, the average number of highlighted terms for snippets is largely reduced. This strategy argues that too many highlighting words can distract users. We need to avoid the AD result of “all in red” tricking users into clicking.
- *Task-Level Highlighting Strategy (S3)*: Considering we have 10 search results for each task, we can merge the 10 highlighted term lists into a task-level highlighted term list. We then use jieba segmentation tool (https://github.com/fxsjy/jieba) to split the words in the task-level highlighted term list into short snippet terms and remove stopwords as well as duplicated snippet terms. We then select snippet terms which are highlighted by at least 5 users and make these terms highlighted in the task's ten snippet results. This strategy assumes that the important information that should be highlighted is relevant only to the query.
- *Result-level Highlighting Strategy (S4)*: For each snippet result, we select out the highlighted words (which may contain several snippet terms) which are highlighted by at least 4 users from its highlighted term list. We make these words highlighted and, in this way, the percentage of highlighted terms mostly equals that of the original highlighting strategy. This strategy argues that important information that should be highlighted is relevant not only to the query, but also to the search result.

We propose the reduced highlighting strategy in order to study the effect of the number of highlighted snippet terms because the original highlighting strategy may highlight too much snippet terms, which may confuse the users. Besides, we also propose the task-level and result-level highlighting strategies in order to find out whether we can select a more reasonable set of highlighted terms.

### 3.3. Task Organization

Considering that we proposed three highlighting strategies (reduced highlighting, task-level highlighting and result-level highlighting) in Section 3.3 besides the original highlighting strategy adopted by Google, we recruited 36 participants and divided them into 3 groups. Each group of participants will finish 12 search tasks designed with the original highlighting strategy and 12 search tasks designed with one specific proposed highlighting strategy in Section 3.3. We adopted a Graeco-Latin square design and randomized sequence order to ensure that search tasks with different highlighting strategies were shown to users with the same opportunity. In this way, we can collect six users' behavior data for each task designed with the original highlighting strategy and six users' behavior data for the same task designed with the corresponding proposed highlighting strategy in each participant group.

To study the effect of highlighted snippet terms, we construct an experimental search engine with the selected search tasks to collect user behavior data on SERPs generated with different highlighting strategies proposed in Section 3.3. With this system, users' interaction behavior logs while completing search tasks are recorded, including eye movements and mouse click-through information.

**Figure 3:**
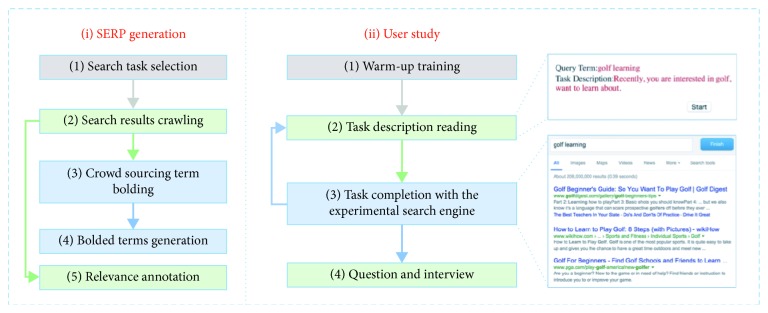
Data collection procedure.

The entire experiment procedure is shown in Figure 3. Before the experiment, each participant should first go through a calibration process as required by the eye tracker to make sure that reliable eye movement information is collected. The eye tracker in our work is Tobii X2-30 with its default parameter settings. Each participant will first finish two warm-up search tasks before the actual tasks to make sure he/she is familiar with the experiment procedure. Before each search task, the participants were required to first go through the search queries and corresponding task descriptions to avoid unnecessary ambiguity. Then, he/she will be guided to a predesigned SERP where snippet terms are highlighted with different highlighting strategies. The participant should examine the search results provided by our system and click a button on the top right corner to end the task and go to the next search task either if the search goal is completed or he/she becomes disappointed with the results. During the participant's search process, his/her mouse click-through data were logged by injected JavaScript on the SERPs, and eye movement information is also logged by the eye tracker. Each participant was required to complete 24 search tasks within 90 minutes and after that, we will ask them some simple questions regarding the queries to make sure they finished the search tasks carefully. We also conducted an interview with some participants after the whole experiment and collect user feedback about the snippet term highlighting strategies.

Among the 36 participants, there are 12 female students and 24 male students (each group was composed of 4 females and 8 males). All participants are first-year undergraduate students from a university with a variety of self-reported search engine utilization experiences. Their majors vary in economics, aesthetics, law, and social science. We did not invite computer science or electrical engineering students because they may be too familiar with search engines and cannot represent ordinary search engine users.

### 3.4. External Annotation

To make a deep analysis of the effect of different term highlighting strategies, we also recruited four professional assessors from a commercial search engine company to label 4-point-scaled relevance scores for all query-result pairs used in our experiment. Each result relevance is judged by four professional assessors and the KAPPA coefficient of their annotation is 0.48, which can be characterized as a moderate agreement according to Cohen [39]. We use these relevance scores to calculate cumulative gains (CG) and discounted cumulative gains (DCG) in later sections to study the effect of highlighted terms on user engagement.

## 4. User Study

In this section, we try to compare different term highlighting strategies with the collected data in a benefit-cost framework. We first show that among the original highlighting strategy and the three strategies proposed in Section 3.3, the result-based highlighting strategy may be the best for users. Then, we conduct a detailed analysis to show how users benefit from the result-based highlighting strategy across different search tasks.

### 4.1. Term Highlighting Strategies

According to the existing researches on the understanding of users' search interaction process [40–42], user engagement may be affected by the benefit they obtain from the SERP and the cost during the search process. So, we also try to analyze the differences between different highlighting strategies following the benefit-cost framework.

The evaluation metrics we use in this section are shown in Table 3. Metrics that start with “C-” are based on click-through features while those start with “E-” are based on eye movement information. The examination threshold is set as 200 milliseconds in our work, which is recommended by previous studies [43, 44]. CG and DCG are metrics to evaluate search benefit while others are adopted to measure user effort. Note that we exclude the time spent on landing pages in DT because we want to focus on the effect of highlighted snippet terms on user engagement on SERPs. These metrics are widely used to measure search benefit and cost in previous related studies [38, 40].

We first compare the effectiveness of different highlighting strategies based on click-through information, and the results are shown in Table 4. We use the default query terms highlighting strategy of Google (S1) as a baseline and only report the differences between the proposed strategies and default one to respect the proprietary nature of the baseline highlighting strategy. Table 4 shows a number of interesting findings:

1. Task-level highlighting strategy (S3) does not bring significant difference over the baseline strategy except that it results in a significant decline of dwell time. This may be because the additional terms beyond query terms used by S3 are a little but not much and increase highlighting terms quantity, which increase search costs.
2. Reduced highlighting strategy (S2) as well as result-level highlighting strategy (S4) are significantly different from the baseline strategy from the perspectives of both benefit and cost. S2 brings significant decline in search cost, which may indicate that too much highlighted terms may not be helpful for users and a small number of long highlighted snippet terms can help them to locate useful information more efficiently. This phenomenon is in line with the findings in Section 3.1. S4 also results in a remarkable decline in search cost, which may indicate that the highlighted terms generated by this method are good quality and are helpful for users during search process.
3. Both the S2 and S4 also bring significant decline in C-CG and C-DCG, which is a bit surprising. But more detailed analysis shows that some highlighted terms generated by these strategies are good enough to be the direct answer to the search task. Therefore, it is unnecessary for users to click these highly relevant results, which may lead to the drop-in search benefit.

Although the results in Table 3 show that both S2 strategy and S4 strategy show improvement over the baseline method, we choose S4 strategy to make a further analysis in next sections because in this way, the ratio of highlighted terms is nearly the same as S1 strategy. So, we can focus our attention on the quality rather than the number of highlighted terms.

### 4.2. Benefit-Cost Analysis

We try to make a further analysis to investigate the differences between the result-level highlighting strategy and the original highlighting strategy based on both click-through information and eye movement information. We also try to investigate the effectiveness of our proposed result-level highlighting strategy on different search tasks. As discussed in Section 3.1, there are three types of search tasks in our work, namely, NA, IN, and TR. Inspired by user feedback and previous findings in Sections 3.1 and 4.1, we also divide our search tasks into two groups by whether there exists instant answers (IA) in the snippet. We first investigate the effect of different highlighting strategies on search cost in these different search tasks, and the results are shown in Table 5.

Table 5 shows that our proposed result-level highlighting strategy can reduce search cost significantly in informational search tasks and transactional tasks. It cannot reduce the search cost in navigational tasks, which may be due to the fact that in such tasks, the target results are usually easy to find; thus, there will be no significant differences between different snippet term highlighting strategies. It is worth noting that the result-level-based method brings remarkable decline in search cost in tasks with instant answers and no significant change for those tasks without instant answers, which may further confirm that our proposed highlighting strategy can help reduce search cost significantly if there exist instant answers on SERPs.

We also try to make an analysis from the perspective of search benefit, and the results are shown in Table 6. We can see that the proposed highlighting strategy results in significant decline in informational tasks and the difference is over 20% in tasks with instant answers, which indicates that snippet terms with helpful information are highlighted with our strategy and thus the users do not need to click or examine so many results. We also take the search task shown in Figure 3 as an example, which is an informational task and also a task with instant answers. The heatmap shows that users can locate the answers to the search task in a very short time without clicking or examining too many results, which will lead to a decrease in both search cost and search benefit.

Findings in this section show that both reduced highlighting strategy and result-level highlighting strategy outperform the original highlighting method adopted by Google, which means fewer and longer highlighted terms may be more helpful to users. Also, snippet terms which can provide instant answers to the search task are so important that they should be highlighted. Based on a benefit-cost framework, we also show that our proposed result-level highlighting strategy can bring the most significant improvement for informational search tasks and the tasks with instant answers.

## 5. Automatic Highlighting Method

In this section, we try to propose a method to highlight snippet text automatically. We first introduce the prediction method and the feature sets used in our work. Then we show the prediction results of automatically snippet text highlighting. We show to what extent we can suggest highlighted terms for a given SERP automatically and compare the performance of different feature sets across different types of search tasks. We also conduct a feature analysis to explore the contribution of different features in automatically snippet term highlighting.

### 5.1. Methodology

We formalize the term highlighting task as a supervised sequence labeling problem. We use jieba segmentation tool to split the snippet text into several terms and then use specified algorithms to automatically predict which snippet term should be highlighted. The learning algorithm selected in the prediction process is conditional random fields (CRF), which is one of the most popular models for structured learning and sequence labeling problems [45].

Existing studies and our experiments in the previous sections highlighted various factors that can affect user engagement with result snippets. Therefore, we take comprehensive information including queries, Wikipedia knowledge as well as search recommendations into consideration when developing features that can be used for automatically snippet text highlighting. The whole list of features is provided in Table 7 and they can be summarized in three groups:

  (★) Exact match-based: These features are adopted to measure whether there exists exact match between the snippet term and query, Wikipedia knowledge, Baidu Baike (a Chinese online encyclopedia, http://baike.baidu.com/), and search recommendations.
  (■) Similarity-based: These features use several distance-based methods as well as tf-idf values to measure the similarity between the snippet term and the corresponding information.
  (◆) Word embedding-based: These features are generated based on word embedding methods (https://code.google.com/p/word2vec/). We use vectors to represent words and calculate various kinds of features. These word vectors are trained based on SogouT dataset (http://www.sogou.com/labs/dl/t-e.html).

With the features described in Table 7, we implement a CRF model based on the python-crfsuite toolkit (https://github.com/tpeng/python-crfsuite) for automatic snippet term highlighting. The dataset in Section 3 is adopted in the prediction experiment with five-fold cross validation and the ideal list of highlighted terms labelled by crowdsourcing workers is used as the ground truth. We should note that this is a nontrivial task as it is an imbalanced learning problem. There are only 8.2% of the snippet terms that are highlighted by users (887 highlighted ones out of 10789 snippet terms in total).

### 5.2. Results

Considering that the standard CRF model implemented based on crfsuite aims to the achieve the best overall performance on predicting both highlighted terms and unhighlighted terms, our main focus should be on the highlighted ones. The effectiveness of CRF model on predicting highlighted snippet terms may be affected because of the imbalance of the dataset. Therefore, based on the predicted probability by CRF model, we test different thresholds to see when we can achieve the best performance on predicting highlighted terms. As shown in Table 8, we report the best precision, recall, and F1-score results on predicting highlighted snippet terms after testing different probability thresholds. Accuracy in the second column of Table 8 is calculated based on all snippet terms, including highlighted ones and unhighlighted ones.

The results in Table 8 show a number of interesting findings:

1. Among the three groups of feature sets, exact match-based features perform worst while similarity-based and word embedding-based features perform much better. This may indicate that users may need more comprehensive information to help with their interaction process. The exact match-based features are effective for finding highlighted query terms but not good at identifying whether a snippet term which is not in the query should be highlighted.
2. We can achieve a slight improvement in F1-score if we combine two feature groups together, except when we combine the exact match-based features and word embedding-based features. This may be because the predicting model runs into an overfitting problem if these two groups of features are combined. If we adopt all three feature groups for term highlighting prediction, we can achieve the best performance, which is significantly better than the prediction results of other feature groups.
3. Although the F1-score achieved by our prediction model is comparatively low, the prediction accuracy on both highlighted terms and unhighlighted terms is around 0.9. This again reflects the fact that predicting highlighted terms is a nontrival task as the dataset is quite unbalanced.

Table 9 shows a further comparison of the prediction results over different search tasks and highlighted terms. All three feature sets are incorporated into the prediction model because in this case, the model performs best as shown in Table 8.

Table 9 shows that our prediction model performs best in NA tasks. This is reasonable because such tasks usually aim to find a particular website which is highly related to the query itself and our features developed based on queries, Wikipedia knowledge as well as search recommendations, which can provide sufficient information. In contrast, IN and TR search tasks often require users to find more comprehensive information, thus suggesting that highlighted terms may become much more difficult. We also test the prediction performance across different snippet terms, namely, query terms (terms which are contained in the search query) and nonquery terms (terms which are not contained in the search query). Table 9 shows that when predicting query terms, the F1-score is up to 78.26% while the F1-score is only 36.92% in the case of nonquery terms. This is not surprising because many features we used are related to the original query, which makes predicting query terms a comparatively easier task. Predicting nonquery terms is a difficult task because of the lack of related information and we will leave it for future work.

We also conduct a feature analysis to explore the contribution of different features in Table 7 on predicting highlighting terms.  Table 10 shows the top 10 features' weights in the trained CRF model. We find that *queryTermW2V* has the largest weight, which belongs to word embedding-based feature group. This feature measures the cosine similarity between the snippet term and the search query, which may be of great help for finding both highlighted query terms and those nonquery terms which are similar to queries from the perspective of word embedding. The second important feature is *ifQueryTerm*, which comes from exact match-based feature group. This feature indicates whether the search query contains the current snippet term and thus is useful for highlighting query terms. The third important feature is from similarity-based feature group, which is *wikiTf*. This feature is calculated by dividing the frequency of the current snippet term in the Wikipedia content by the length of Wikipedia content. It may be useful to find more comprehensive information about the search task rather than the original query, which may help highlighting those nonquery snippet terms.  Table 10 also shows that the top 10 important features include features from all these three feature sets, which indicates that all of them are useful. Meanwhile, word embedding-based features may be the most important because half of the top 10 important features are based on word embedding methods and the most important one is also developed based on word vectors.

## 6. Conclusion

Search result snippet serves a very important role in search interaction process, and the effect of different highlighting strategies has not been pursued. In this paper, we conduct a lab-based study with carefully designed experiments to investigate which snippet terms should be highlighted and how they affect user engagement. We organize a crowdsourcing effort to annotate the snippet terms which are helpful for the search task and develop several snippet term highlighting strategies to compare their effectiveness within a benefit-cost framework. We find that fewer and longer highlighted snippet terms can be helpful for users and the proposed result-level highlighting strategy can help users locate their targets more efficiently, which significantly reduces search cost. We demonstrate that the result-level highlighting strategy can significantly outperform the original highlighting strategy in informational search tasks and those tasks with instant answers. We also propose an automatically snippet term highlighting method with the information from Wikipedia, Baidu Baike, search recommendations, and word embedding, which achieves promising results in highlighting both query terms and nonquery terms in snippet text. Of course, we just verify that different highlighting strategies can affect users' behavior and do not directly distinguish the pros and cons between two different strategies. The future work requires more extensive research on the highlighting strategy evaluation system and automatic evaluation methods. And the interesting directions for future work also include developing more effective automatic term highlighting methods, especially for automatically highlighting snippet terms which are not query terms.

## Figures and Tables

**Table 1:** The 24 queries from the NTCIR IMine task.

Query
ICBC home page
Cui Yongyuan's blog
Live on mango TV
Chinese-English online
Alipay customer service number
Founding members of the AIIB
How about MI 4
Simple hair retrieval method
Anchor of treasure inspect
New year card design
TOYOTA REIZ
Song Mei-ling's date of death
Xiao Muchong home page
301 hospital
QQ free download
Air waybill inquiry
Sub - Crown
Chinese capitalized numbers 1 to 10
Jiangxi Wuyuan
AIDS skin symptoms
Types of hypertensive
Red wine
Golf learning
Chinese Zodiac origin

**Table 2:** Some queries and detailed task explanations.

Class	Query	Task explanation
NA	ICBC home page	You have an ordinary bank card of ICBC and have opened an online bank. You want to visit the home page of ICBC for information about online bank

TR	Live on mango TV	You are a loyal fan of mango TV and a variety show enthusiast. Today is your favorite variety show. You want to watch Mango live for the first time

IN	How about MI 4	Your Samsung mobile phone has broken down, and you want to buy a new smartphone. You want to know how the performance of MI 4 is

**Table 3:** Evaluation metrics in benefit-cost framework.

Metrics	Description
C-CG	Cumulated gain calculated based on users' clicked result list
C-DCG	Discounted cumulated gain calculated based on users' clicked result list
E-CG	Cumulated gain calculated based on users' examined result list
E-DCG	Discounted cumulated gain calculated based on users' examined result list
DT	Search task dwell time, not including the time spend on landing pages
C-RN	Number of clicked results
C-RD	Maximum rank of clicked results
C-SL	Length of clicked result list
E-RN	Number examined results
E-RD	Maximum rank of examined results
E-SL	Length of examined result list

**Table 4:** Comparison between different term highlighting strategies.

	S2 (%)	S3 (%)	S4 (%)
C-CG	▼8.8	↓4.7	▼8.3
C-DCG	▼7.3	↓4.1	▼7.6
DT	▼12.8	▼13.8	▼12.5
C-RN	▼10.5	↓5.5	▼9.1
C-RD	▼17.6	↓0.8	↓9.8
C-SL	▼10.0	↓5.0	▼9.4

Percentage decrease is, respectively, denoted by down arrow and filled triangle; filled triangle indicates t-test; statistical significance at *p* < 0.1 level.

**Table 5:** Search cost comparison across different search tasks.

	NA (%)	IN (%)	TR (%)	w/IA (%)	w/oIA (%)
DT	↑6.3	▼18.7	13.0	▼27.2	▼6.0
C-RN	↓5.0	↓7.8	▼19.0	▼16.3	▼7.2
C-RD	↑26.7	▼13.7	▼17.6	▼28.4	▼4.3
C-SL	5.0	▼8.2	18.6	▼17.5	▼7.2
E-RN	↑19.4	↓9.3	↑24.0	▼20.2	↑2.4
E-RD	*▲*23.7	↓13.7	↑5.6	▼34.2	↑2.6
E-SL	↑44.3	↓11.1	↑24.0	↓23.5	↑6.8

Percentage decrease is, respectively, denoted by down arrow and down filled triangle, and percentage increase is, respectively, denoted by up arrow and up filled triangle. Filled triangles indicate *t*-test; statistical significance at *p* < 0.1 level.

**Table 6:** Search benefit comparison across different search tasks.

	NA (%)	IN (%)	TR (%)	w/IA (%)	w/oIA (%)
C-CG	↓8.2	▼9.1	▼22.4	▼21.7	▼8.3
C-DCG	↑6.2	▼4.8	↓20.9	↓7.0	▼7.8
E-CG	↑15.6	▼10.6	↑14.6	▼20.4	↑0.7
E-DCG	↑14.3	▼17.6	↓18.2	↓26.4	↓10.6

Percentage decrease are, respectively, denoted by down arrow and down filled triangle, and percentage increase is, respectively, denoted by up arrow. Filled triangle indicates t-test; statistical significance at *p* < 0.1 level.

**Table 7:** Features used for automatically snippet text bolding.

Feature	Group	Description
*ifQueryTerm*	★	Whether the snippet term is a query term
*ifResulttitle*	★	Whether the snippet term is a term in the result title
*ifInWiki*	★	Whether the snippet term appears in the Wikipedia content of the query
*wikiCount*	★	Frequency of the snippet term in the Wikipedia content of the query
*ifInBaidu*	★	Whether the snippet term appears in the Baidu Baike content of the query
*baiduCount*	★	Frequency of the snippet term in the Baidu Baike content of the query
*ifSearchRec*	★	Whether the snippet term appears in the search recommendations of the query
*searchRecCount*	■	Frequency of the snippet term in the search recommendations of the query
*queryTermJaccard*	■	Jaccard distance between the snippet term and query
*queryTermEdit*	■	Edit distance between the snippet term and query
*searchResultsOverlap*	■	Number of shared results of the search result lists obtained by submitting the snippet term and query to commercial search engine
*wikiTfIdf*	■	Tf-idf value of the snippet term in the Wikipedia corpus (Tf value is calculated as the frequency of the snippet term in the Wikipedia content of the query Wikipedia contents of all the queries used in our experiment are used to calculate the Idf value)
*baiduTfIdf*	■	Tf-idf value of the snippet term in the Baidu Baike corpus. Similar to wikiTfIdf
*searchRecTfIdf*	■	Tf-idf value of the snippet term in the search recommendation corpus. Similar to wikiTfIdf
*termTermW2V*	◆	Cosine similarities between the snippet term vector and query term vectors (if the query is composed of *n* terms after segmentation, then we will get *n* cosine similarities)
*termTermProW2V*	◆	Average, top 3 average, medium, maximum and minimum of termTermW2V
*queryTermW2V*	◆	The cosine similarity between the query vector and snippet term vector (if the query is composed of *n* terms after segmentation, we use the average vector of the *n* term vectors to be the query vector)
*resultTitleTermW2V*	◆	The cosine similarity between the title vector and snippet term vector (if the title is composed of *n* terms after segmentation, we use the average vector of the *n* term vectors to be the title vector)
*searchRecW2V*	◆	The cosine similarities between the snippet term and the search recommendation corpus. Similar to queryTermProW2V

**Table 8:** Comparison of different feature sets for automatic snippet text highlighting.

Features	*P* (%)	*R* (%)	*F*1 (%)
★	22.50	99.22	36.68
■	60.60	80.60	69.18
◆	59.85	85.15	70.29
★■	63.00	84.89	72.32
★◆	23.55	**99.77**	38.11
■◆	64.50	79.56	71.24
★■◆	**65.85**	86.32	**74.71**

**Table 9:** Prediction performance across different search tasks and snippet terms.

Search tasks	Highlighting terms	*F*1 (%)
NA tasks	All terms	**84.00**
IN tasks	All terms	69.75
TR tasks	All terms	64.50
All tasks	Query terms	**78.26**
All tasks	Nonquery terms	36.92

**Table 10:** Feature weights in CRF model.

Feature	Weight (%)	Group
*queryTermW2V*	72.30	◆
*ifQueryTerm *	52.92	★
*wikiTf*	50.25	■
*termTermW2V*	37.50	◆
*averageTermTermW2V*	32.25	◆
*queryTermJaccard*	25.95	■
*searchRecCount*	25.45	★
*top3AverageTermTermW2V*	25.35	◆
*termTermW2V *	25.20	◆
*wikiTfIdf*	24.15	★

## Data Availability

The data used to support the findings of this study are available from the corresponding author upon request.
